# Supplementary material for: Between Resilience and Agency: A Systematic Review of Protective Factors and Positive Experiences of LGBTQ+ Students
Source: Healthcare (Basel). 2023 Jul 23;11(14):2098. doi: 10.3390/healthcare11142098 (PMC10379181; doi:10.3390/healthcare11142098)
Supplement: Supplementary file 1 [file healthcare-11-02098-s001.zip › healthcare-2474793-supplementary.pdf]

Table S1. Data collection table

First author/year	Title	Location	*N* LGBTQ+ students (age >14<19)	Methodology
Arnarsson et al., 2015	Suicidal risk and sexual orientation in adolescence: A population-based study in Iceland	Iceland	57 lesbian, gay and bisexual (LGB)	Survey<br>Quantitative analysis
Colvin et al., 2019	School climate & sexual and gender minority adolescent mental health	USA	240 sexual/gender minority (SGM)	Survey<br>Quantitative analysis
Craig et al., 2014	School-based groups to support multiethnic sexual minority youth resiliency: Preliminary effectiveness	Canada	263 sexual minority (SM)	Quantitative analysis/Pretest and posttest programme assessment
Day et al., 2019	Safe and supportive schools for LGBT youth: Addressing educational inequities through inclusive policies and practices	USA	5598 LGB and 1362 trans (T)	Quantitative analysis (students and principals' reports)
De Pedro et al., 2017	School protective factors and substance use among lesbian, gay, and bisexual adolescents in California public schools	USA	21,953 LGB	Survey<br>Quantitative analysis
Eisenberg et al., 2017	Risk and protective factors in the lives of transgender/gender nonconforming adolescents	USA	2,168 T and gender non-conforming	Survey<br>Quantitative analysis
Eisenberg et al., 2019	Emotional distress, bullying victimization, and protective factors among transgender and gender diverse adolescents in city, suburban, town, and rural locations	USA	2,168 TGD	Survey<br>Quantitative analysis (covariance models)
Erhard et al., 2016	The schooling experience of lesbian, gay, and bisexual youth in Israel: Falling below and rising above as a matter of social ecology	Israel	20 LGB	Interviews<br>Exploratory research<br>Qualitative (thematic analysis)
Evans et al., 2021	It was just one less thing that I had to worry about': Positive experiences of schooling for gender diverse and transgender students	Australia	3 T	Interviews<br>Qualitative analysis
Francis et al., 2020	Troubling the discourse of the victimization of queer youth in Icelandic and South African education	Iceland & South Africa	38 queer (Q) (19 from Iceland, 19 from South Africa)	In-depth interviews<br>Qualitative analysis
Freitas et al., 2017	Mecanismos de proteção perante a vitimização por pares e a discriminação	Portugal	84 LGB	Survey<br>Quantitative analysis
Gower et al., 2018	School practices to foster LGBT-supportive climate: Associations with adolescent bullying involvement	USA	1931 LGBQ	Mixed method<br>Quantitative analysis<br>Reports analysis
Gower et al., 2018[2]	Supporting transgender and gender diverse youth: Protection against emotional distress and substance use	USA	2,168 T, genderqueer, genderfluid, or questioning their gender	Survey<br>Quantitative data
Greytak et al., 2013	Putting the 'T' in 'resource': The benefits of LGBT-related school resources for transgender youth	USA	6,174 LGBT and 409 T	Survey<br>Quantitative analysis
Higa et al., 2014	Negative and positive factors associated with the well-being of lesbian, gay, bisexual, transgender, queer, and questioning (LGBTQ) youth	USA	68 LGBTQ	Focus groups<br>Interviews<br>Qualitative analysis
Hillier et al., 2019	Risk, resilience, resistance and situated agency of trans high school students	USA	22 T	Interviews<br>Qualitative<br>Thematic analysis

Ioverno et al., 2016	The protective role of gay–straight alliances for lesbian, gay, bisexual, and questioning students: A prospective analysis	USA	327 LGBQ	Quantitative analysis<br>Longitudinal data
Konishi et al., 2014	Still a target: Sexual diversity and power of caring	Canada	810 LGB	Survey<br>Quantitative analysis
Kosciw et al., 2015	Reflecting resiliency: Openness about sexual orientation and/or gender identity and its relationship to well-being and educational outcomes for LGBT students	USA	7,816 LGBT	Survey<br>Quantitative analysis
Leonard, 2022	'It was probably one of the best moments of being trans, honestly!': Exploring the positive school experiences of transgender children and young people	UK	3 T	Focus groups<br>Interviews<br>Interpretative Phenomenological Analysis
Lessardet al., 2020	Gay–straight alliances: A mechanism of health risk reduction among lesbian, gay, bisexual, transgender, and questioning adolescents	USA	17,112 LGBTQ	Survey<br>Quantitative analysis
McCormack et al., 2012	The positive experiences of openly gay, lesbian, bisexual and transgendered students in a christian sixth form college	UK	4 LGBT	Interviews<br>Etnographic participant observation<br>Qualitative analysis
McCormick et al., 2015	Gay–straight alliances: Understanding their impact on the academic and social experiences of lesbian, gay, bisexual, transgender, and questioning high school students	USA	36 LGBTQ	Interviews<br>Qualitative analysis
McGowan et al., 2022	Living your truth: Views and experiences of transgender young people in secondary education	Scotland	10 T	Interviews<br>Qualitative<br>Thematic analysis
McKay et al. 2019	Suicide etiology in youth: Differences and similarities by sexual and gender minority status	USA	175 SGM	Quantitative analysis<br>Secondary data
Mintz et al., 2021	Supporting sexual minority youth: Protective factors of adverse health outcomes and implications for public health	USA	580 LGBQ	Survey (across four semesters<br>Quantitative analysis
Morris et al., 2014	The changing experiences of bisexual male adolescents	UK	15 male B	Interviews<br>Critical interpretative approach<br>Qualitative analysis
Msibi 2012	I'm used to it now': Experiences of homophobia among queer youth in South African township schools	South Africa	8 black LGQ	Life narratives interviews<br>Qualitative
O'Brien et al., 2022	Holding the space: Individual- and group-level factors predicting member retention in gender-sexuality alliances	USA	340 SM	Quantitative survey data analysis
Odenbring et l, 2021	Trapped between: 'coming out' and forced to stay closeted	Sweden	1 female B	In-depth interviews with one key informant<br>Qualitative
Parmar et al., 2022	Investigating protective factors associated with mental health outcomes in sexual minority youth	USA	1943 SMY	Survey Cohort study<br>Quantitative analysis
Phillips et al., 2020	Engagement with LGBTQ community moderates the association between victimization and substance use among a cohort of sexual and gender minority individuals assigned female at birth	USA	400 SGM assigned female at birth	Survey<br>Quantitative analysis
Poteat et al., 2015	Contextualizing gay-straight alliances: Student, advisor, and structural factors related to positive youth development among members	USA	146 GSA members	Survey<br>Quantitative analysis

Poteat et al., 2016	Promoting youth agency through dimensions of gay–straight alliance involvement and conditions that maximize associations	USA	203 LGBQ	Survey<br>Quantitative analysis
Poteat et al., 2018	Gay-Straight Alliance involvement and youths' participation in civic engagement, advocacy, and awareness-raising	USA	205 LGB (and 87 heterosexual) members of GSA	Survey<br>Quantitative analysis
Poteat et al., 2020	Membership experiences in gender-sexuality alliances (GSAs) predict increased hope and attenuate the effects of victimization	USA	366 members of GSA	Two wave survey<br>Quantitative analysis
Price et al., 2019	Teacher relationships and adolescents experiencing identity-based victimization: What matters for whom among stigmatized adolescents	USA	216 SMY	Survey<br>Quantitative analysis
Proulx et al., 2019	Associations of lesbian, gay, bisexual, transgender, and questioning–inclusive sex education with mental health outcomes and school-based victimization in U.S. high school students	USA	6,204 LGBTQ)	Survey<br>Quantitative analysis
Ross-Reed et al., 2019	Family, school, and peer support are associated with rates of violence victimization and self-harm among gender minority and cisgender youth	USA	1107 GM	Survey<br>Quantitative analysis
Russell et al., 2013	Risk and protective factors for suicidal thoughts among sexual minority youth: Evidence from the add health study	USA	916 SMY	Longitudinal study<br>Quantitative analysis
Russell et al., 2020	Gender diversity and safety climate perceptions in schools and other youth-serving organisations	Australia	27 gender diverse	Survey<br>Quantitative analysis
Schimmel-Bristow et al., 2018	Youth and caregiver experiences of gender identity transition: A qualitative study	USA	15 T	Interviews<br>Thematic analysis<br>Qualitative
Schimmel-Bristow et al., 2019	Youth and caregiver experiences of gender identity transition: A qualitative study	USA	15 T	Interviews<br>Focus groups<br>Theoretical thematic analysis
Snapp et al., 2015	Students' perspectives on LGBTQ-inclusive curriculum	USA	26 GSA members	Focus groups<br>Qualitative
Standley et al., 2021	Intersectionality, social support, and youth suicidality: A socioecological approach to prevention	USA	718 SMY	Survey<br>Quantitative analysis
Szlyk et al., 2021	Narratives of suicidality, alternative education, and resiliency: Implications for social work practice and research	USA	1 T and 1 B	Interviews<br>Qualitative<br>Narrative analysis
Taliaferro et al., 2018	Connections that moderate risk of non-suicidal self-injury among transgender and gender non-conforming youth	USA	2,168 TGNC (transgender and gender non conforming)	Survey<br>Quantitative analysis
Taliaferro et al., 2019	Risk and protective factors for self-harm in a population-based sample of transgender youth	USA	1,635 TGBC	Survey<br>Quantitative analysis
Toomey et al., 2013	Gay-straight alliances, social justice involvement, and school victimization of Lesbian, gay, bisexual, and queer youth: Implications for school well-being and plans to vote	USA	230 LGBQ	Survey<br>Quantitative analysis
Toomey et al., 2016	Mindfulness as a coping strategy for bias-based school victimization among Latina/o sexual minority youth	USA	111 SM Latina/o SM	Survey<br>Quantitative analysis
Toomey et al., 2018	Coping with sexual orientation–related minority stress, USA 2018	USA	245 LGB (aged 21-25 years - it concerned their youth experiences)	Survey<br>Quantitative analysis

Toomey et al., 2019	Are developmental assets protective against suicidal behaviour? Differential associations by sexual orientation	USA	11,459 LGB	Survey<br>Quantitative analysis
van Bergen et al., 2014	'Their words cut me like a knife': Coping responses of Dutch lesbian, gay and bisexual youth to stigma	The Netherlands	30 LGB	Interviews<br>Qualitative analysis
Vigna et al., 2018	Does self-compassion covary with minority stress? Examining group differences at the intersection of marginalized identities	USA	396 SGM	Survey<br>Quantitative analysis
Watson et al., 2017	Disordered eating behaviors among transgender youth: Probability profiles from risk and protective factors	Canada	323 T	Survey<br>Quantitative analysis
Watson et al., 2020	Associations between community-level LGBTQ-supportive factors and substance use among sexual minority adolescents	Canada	2678 SMY	Survey<br>Community and school data<br>Quantitative analysis
Wei et al., 2019	Coming out in Mainland China: A national survey of LGBTQ students	China	132 LGBTQ	Survey<br>Quantitative analysis
Wernick et al., 2013	LGBTQQ youth creating change: Developing allies against bullying through performance and dialogue	USA	137 LGBQ and 35 T/genderqueer members of middle and high school alliances	Pre and post-test surveys<br>Quantitative analysis
Wernick et al., 2014	How theater within a transformative organizing framework cultivates individual and collective empowerment among LGBTQQ youth	USA	8 LGBTQQ	Interviews<br>Qualitative analysis
Whitaker et al., 2016	School-based protective factors related to suicide for lesbian, gay, and bisexual adolescents	USA	356 LGB	Survey<br>Quantitative analysis
Wilkerson et al., 2017	Social support, depression, self-esteem, and coping among LGBTQ adolescents participating in hatch youth	USA	108 LGBTQ	Survey<br>Quantitative analysis
Woolley et al., 2020	Bisexuality, bad girls, and bullying	USA	2 female B and GSA members from one school	Ethnography (interviews, focus groups, classroom recordings)<br>Qualitative analysis
Yang et al., 2014	Flower boys on campus: Performing and practicing masculinity	Taiwan	8 'fem boys'	Ethnography (observations, focus groups, interviews, and document collection)<br>Qualitative analysis
Zeeman et al., 2017	Promoting resilience and emotional well-being of transgender young people: Research at the intersections of gender and sexuality	UK	5 T	Focus group<br>Thematic analysis<br>Qualitative
